# 2-[^18^F]FDG-PET/CT is a better predictor of survival than conventional CT: a prospective study of response monitoring in metastatic breast cancer

**DOI:** 10.1038/s41598-023-32727-w

**Published:** 2023-04-05

**Authors:** Marianne Vogsen, Mohammad Naghavi-Behzad, Frederik Graae Harbo, Nick Møldrup Jakobsen, Oke Gerke, Jon Thor Asmussen, Henriette Juel Nissen, Sara Elisabeth Dahlsgaard-Wallenius, Poul-Erik Braad, Jeanette Dupont Jensen, Marianne Ewertz, Malene Grubbe Hildebrandt

**Affiliations:** 1 Department of Oncology, Odense University Hospital, Kloevervaenget 47, 5000 Odense C, Denmark; 2 Department of Nuclear Medicine, Odense University Hospital, Odense, Denmark; 3 Department of Clinical Research, University of Southern Denmark, Odense, Denmark; 4 OPEN, Odense Patient Data Explorative Network, Odense University Hospital, Odense, Denmark; 5 Centre for Personalized Response Monitoring in Oncology (PREMIO), Odense University Hospital, Odense, Denmark; 6 Department of Radiology, Odense University Hospital, Odense, Denmark; 7 Centre for Innovative Medical Technology, Odense University Hospital, Odense, Denmark

**Keywords:** Breast cancer, Cancer imaging

## Abstract

This study aimed to compare CE-CT and 2-[^18^F]FDG-PET/CT for response monitoring metastatic breast cancer (MBC). The primary objective was to predict progression-free and disease-specific survival for responders vs. non-responders on CE-CT and 2-[^18^F]FDG-PET/CT. The secondary objective was to assess agreement between response categorization for the two modalities. Treatment response in women with MBC was monitored prospectively by simultaneous CE-CT and 2-[^18^F]FDG-PET/CT, allowing participants to serve as their own controls. The standardized response evaluation criteria in solid tumors (RECIST 1.1) and PET response criteria in solid tumors (PERCIST) were used for response categorization. For prediction of progression-free and disease-specific survival, treatment response was dichotomized into responders (partial and complete response) and non-responders (stable and progressive disease) at the first follow-up scan. Progression-free survival was defined as the time from baseline until disease progression or death from any cause. Disease-specific survival was defined as the time from baseline until breast cancer-specific death. Agreement between response categorization for both modalities was analyzed for all response categories and responders vs. non-responders. At the first follow-up, tumor response was reported more often by 2-[^18^F]FDG-PET/CT than CE-CT, with only fair agreement on response categorization between the two modalities (weighted Kappa 0.28). Two-year progression-free survival for responders vs. non-responders by CE-CT was 54.2% vs. 46.0%, compared with 59.1% vs. 14.3% by 2-[^18^F]FDG-PET/CT. Correspondingly, 2-year disease-specific survival were 83.3% vs. 77.8% for CE-CT and 84.6% vs. 61.9% for 2-[^18^F]FDG-PET/CT. Tumor response on 2-[^18^F]FDG-PET/CT was significantly associated with progression-free (HR: 3.49, P < 0.001) and disease-specific survival (HR 2.35, P = 0.008), while no association was found for tumor response on CE-CT. In conclusion, 2-[^18^F]FDG-PET/CT appears a better predictor of progression-free and disease-specific survival than CE-CT when used to monitor metastatic breast cancer. In addition, we found low concordance between response categorization between the two modalities.

**Trial registration:** Clinical.Trials.gov. NCT03358589. Registered 30/11/2017-Retrospectively registered, http://www.ClinicalTrials.gov.

## Introduction

Metastatic breast cancer (MBC) is an incurable disease, but more effective treatments have increased survival and render MBC a chronic disease^1^. Monitoring the effect of treatment is important to ensure that the treatment remains effective or, if not, to allow a rapid change in treatment. Therefore, the choice of diagnostic modality and standardized criteria for response evaluation in this patient group is essential for optimal treatment planning^1–3^.

Although an increasing number of patients receive treatment for MBC, clinical guidelines still make no clear recommendations on which diagnostic modality to choose for monitoring treatment effect^1,4^. Conventional contrast-enhanced CT (CE-CT) is the most commonly used modality in daily clinical practice and clinical trials. CE-CT and the corresponding response evaluation criteria in solid tumors (RECIST 1.1) are methods that assess changes in structural lesions, making it challenging to differentiate between treatment response and tumor progression^5–8^. Consequently, tumor response assessed by CE-CT has been reported to correlate poorly with survival^9^.

2-Deoxy-2-[^18^F]fluoro-d-glucose-positron-emission-tomography/computed tomography (2-[^18^F]FDG-PET/CT) is suggested as an alternative to CE-CT with higher accuracy for diagnosing metastases from breast cancer, especially in the bones and liver^10^. 2-[^18^F]FDG-PET/CT and the corresponding PET response criteria in solid tumors (PERCIST) have shown to be feasible for response monitoring in women with MBC^7,11^ and to correlate better with survival than RECIST 1.1^9,12^. In a recent head-to-head comparison in our group, PERCIST revealed higher response rates than RECIST 1.1 and classified more patients with measurable disease, indicating important implications for clinical trials^9,13^. Furthermore, progression seems to be detected earlier by 2-[^18^F]FDG-PET/CT than CE-CT, allowing earlier treatment alterations and a potential survival benefit for patients with MBC^13,14^.

We still need prospective studies comparing 2-[^18^F]FDG-PET/CT with CE-CT for the prediction of progression-free survival (PFS) and disease-specific survival (DSS). In this study, we compare CE-CT and 2-[^18^F]FDG-PET/CT for response monitoring metastatic breast cancer. The primary objective was to investigate the prediction of progression-free and disease-specific survival for responders vs. non-responders with RECIST 1.1 and PERCIST, respectively. The secondary objective was to assess the agreement between response categorization for the two sets of response criteria.

## Methods

### Study design and patients

In this prospective observational study, the response to first-line treatment for MBC was compared for CE-CT and 2-[^18^F]FDG-PET/CT, with patients serving as their own control.

Patients were identified from a previously reported study population from which data on time-related detection of progression, measurable disease, and distribution of response categories have been published separately^13^. The study was registered at Clinical.Trials.gov (NCT03358589), and the results were reported using the STROBE guideline^15^.

Inclusion criteria were a diagnosis with de novo or recurrent MBC and eligibility for systemic oncological treatment. Signed consent was obtained from enrolled patients after written and oral information of the study. Patients were excluded from the study if MBC was not verified by biopsy or if they departed or died before the first follow-up scan. Data were collected from medical records, pathology reports, monitoring images, and scan reports.

### Imaging and response evaluation criteria

Before initiating first-line treatment, patients had a combined 2-[^18^F]FDG-PET/CT performed with CE-CT at baseline to diagnose MBC^16,17^. 2-[^18^F]FDG-PET/CT and CE-CT were performed simultaneously for each follow-up scan, but treatment decisions were based on CE-CT without knowledge of 2-[^18^F]FDG-PET images.

2-[^18^F]FDG-PET/CT was performed according to the European Association of Nuclear Medicine guideline from the top skull to mid-thigh 60 ± 5 min p.i. with intravenous injection of 4 MBq 2-[^18^F]FDG per kg bodyweight^18^. Patients fasted at least 4 h before 2-[^18^F]FDG injection, and blood sugar levels were measured routinely. 2-[^18^F]FDG-PET/CT scans were assessed according to the PERCIST one-lesion guideline^19^ by introducing the nadir level of the standardized uptake value normalized by lean body mass in the hottest metastatic lesion (SULpeak) if measurable disease was present^11^. Otherwise, a visual assessment was used for response categorization. Tumor response on follow-up scans was categorized into one of four groups: complete metabolic response (CMR), partial metabolic response (PMR), stable metabolic disease (SMD), or progressive metabolic disease (PMD).

CE-CT was performed as part of 2-[^18^F]FDG-PET/CT and assessed without prior knowledge from 2-[^18^F]FDG-PET images according to RECIST 1.1^5^. A visual assessment was used in cases of no measurable disease. Tumor response on follow-up scans was categorized into one of four groups: complete response (CR), partial response (PR), stable disease (SD), or progressive disease (PD).

Further details on imaging techniques, response evaluation criteria, and response categorization appear elsewhere^13^.

### Statistics

Continuous data are presented using the median (range). Frequencies and respective percentages are given for categorical variables.

The primary endpoints were PFS and DSS. Median 2-year and 4-year PFS and DSS were evaluated with 95% confidence intervals (95% CI). Progression was defined as progression leading to change in first-line treatment, and follow-up was extended from the previous report until 20 April 2022. PFS was defined as the time from baseline scan until disease progression or death from any cause, and DSS from baseline scan until breast cancer-specific death. The date of the last clinical follow-up was considered a censoring event for both PFS and DSS. During initial follow-up, treatment response was assessed by CE-CT with 2-[^18^F]FDG-PET/CT blinded for clinical evaluation. After the end-of-trial by November 2020, 2-[^18^F]FDG-PET/CT scans were unblinded, and patients were monitored prospectively by 2-[^18^F]FDG-PET/CT as a clinical routine in our institution.

For predicting PFS and DSS, treatment response was dichotomized into responders vs. non-responders and progression vs. non-progression (disease control rate) for CE-CT and 2-[^18^F]FDG-PET/CT, respectively. Responders were defined as PR/CR for CT and PMR/CMR for 2-[^18^F]FDG-PET/CT, whereas non-responders were defined as SD/PD and SMD/PMD for CE-CT and 2-[^18^F]FDG-PET/CT, respectively (Table 1). The disease control rate was defined as all response categories other than PD/PMD. Kaplan–Meier survival curves, including risk tables, were used for visualization^20^, and a Cox regression model was conducted to investigate the prediction of PFS and DSS by the modalities, indicating the difference between responders vs. non-responders by a hazard ratio (HR).

**Table 1:** Response categorization on CE-CT and 2-[^18^F]FDG-PET/CT for the first follow-up scan in 87 patients.

Response category	2-[^18^F]FDG-PET/CT N (%)		CE-CT N (%)	
Total	87 (100)		87 (100)	
Responders				
C(M)R	8 (9.2)	66 (75.9)	2 (2.3)	25 (28.7)
P(M)R	58 (66.7)		23 (26.4)	
Non-responders				
S(M)D	13 (14.9)	21 (24.1)	59 (67.8)	62 (71.3)
P(M)D	8 (9.2)		3 (3.5)	

*C(M)R* complete (metabolic) response, *P(M)R* partial (metabolic) response, *S(M)D* stable (metabolic) disease, *P(M)D* progressive (metabolic) disease.

For agreement analysis, response categories were assessed separately and dichotomized into responders and non-responders, as mentioned above^13^. Concordance between 2-[^18^F]FDG-PET/CT and CE-CT-based response categories was calculated using Cicchetti–Allison-weighted kappa statistics. A Kappa of 0.81–1.00 was considered as almost perfect agreement, 0.61–0.80 as substantial agreement, 0.41–0.60 as moderate agreement, 0.21–0.40 as fair agreement, 0–0.20 as slight agreement, and < 0.00 as poor agreement^21^. A separate agreement rate was calculated for patients with bone-only metastasis. The significance level was set at 0.05. All statistical analyses were conducted with STATA/IC (version 16.1, StataCorp, College Station, USA).

### Ethical approval

The study was performed in line with the principles of the Declaration of Helsinki, approval was granted by the Danish Ethics Committee in Southern Denmark (S-20170019), and patients signed a consent statement. The study was registered at Clinical.Trials.gov (NCT03358589) and data were stored in the secure systems REDCap (Research Electronic Data Capture) and SharePoint. The results were reported using the STROBE guideline^15^.

### Consent to participate

Informed consent was obtained from all individual participants included in the study.

## Results

The median age of the 87 enrolled patients was 72.7 (41.1–89.4) years, and most patients had estrogen receptor (ER) positive disease with normal expression of human epidermal growth factor receptor (HER2), receiving endocrine therapy ± cyclin-dependent kinase 4/6 inhibitor as first-line therapy. According to the baseline scan, 65/87 (74.7%) of the patients had bone metastases, with bone-only metastatic disease in 23/87 (26.4%) patients. A flowchart and table of patient baseline characteristics appear elsewhere^13^.

The median follow-up time was 36.1 (2.89–55.0) months, and the median PFS and DSS were 23.5 (95% CI 15.8–35.0) and 43.2 (95% CI 33.9–∞) months, respectively. The median PFS was nearly twice as high for patients with bone-only disease (42.9, 95% CI 22.0–∞ months), with no difference in DSS compared with the total cohort (43.8, 95% CI 36.1–∞ months).

### Response categorization and survival

Response categorization according to CE-CT and 2-[^18^F]FDG-PET/CT on the first follow-up scans are shown in Table 1. More patients were classified as responders (CMR + PMR) on 2-[^18^F]FDG-PET/CT than on CE-CT on the first follow-up scan, while CE-CT revealed more stable disease. The distribution of response categories for all follow-up scans is seen in Table 2.

**Table 2:** Distribution and agreement between response categorization according to CE-CT and 2-[^18^F]FDG-PET/CT in 87 patients with 517 follow-up scans.

	CE-CT				
	CR	PR	SD	PD	Total
2-[18F]FDG-PET/CT					
CMR	**27**	84	36	2	149
PMR	5	**107**	79	5	196
SMD	0	4	**32**	0	36
PMD	8	45	43	**40**	136
Total	40	240	190	47	**517**
Agreement 39.9%		Weighted Kappa 0.28		Std. error 0.03	

Significant values are in [bold].

*C(M)R* complete (metabolic) response, *P(M)R* partial (metabolic) response, *S(M)D* stable (metabolic) disease, *P(M)D* progressive (metabolic) disease.

**Figure 1:**
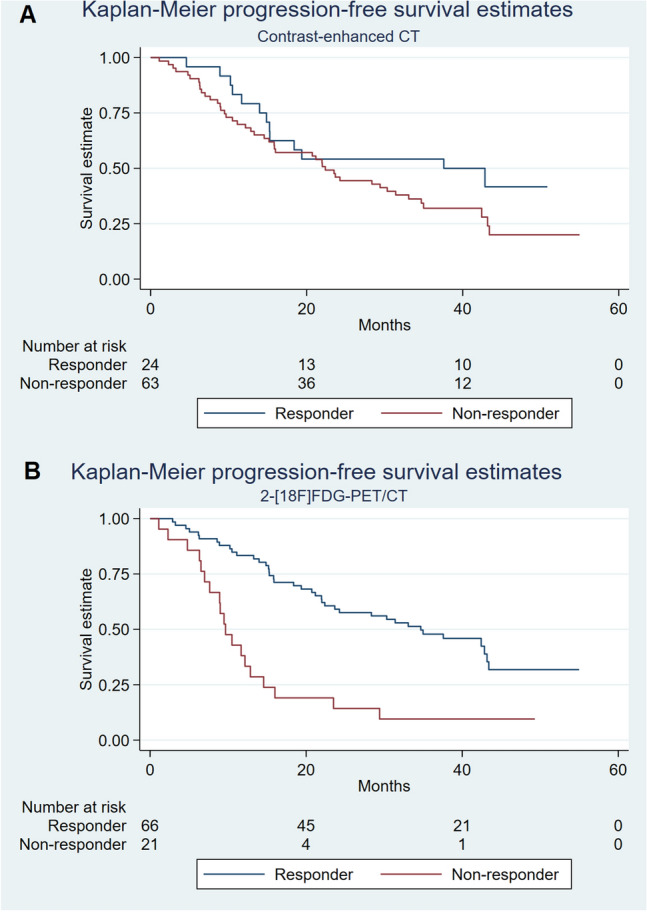
Kaplan–Meier plot of progression-free survival for responders and non-responders on (**A**) CE-CT and (**B**) 2-[^18^F]FDG-PET/CT on the first follow-up scans.

**Figure 2:**
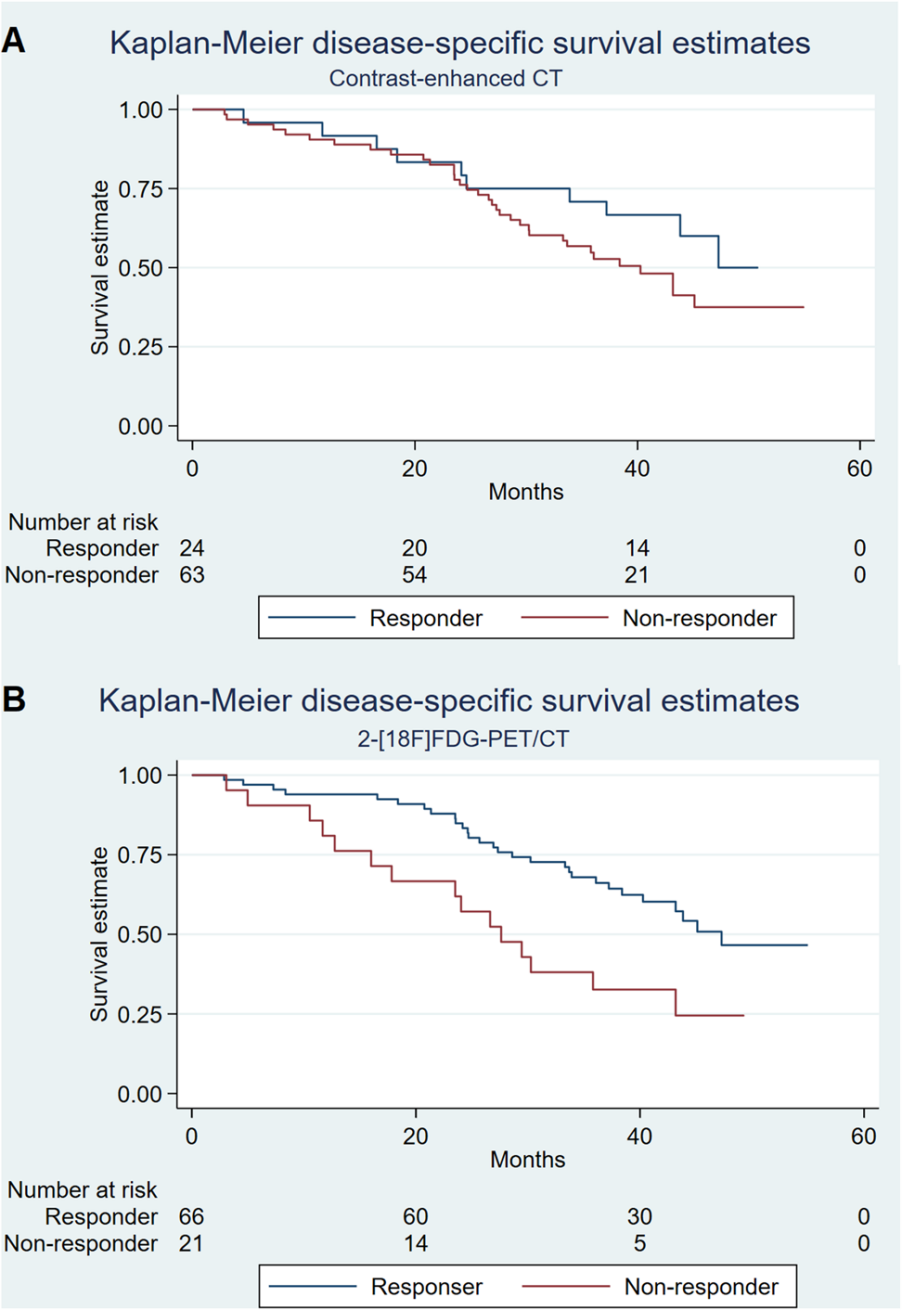
Kaplan–Meier plot of disease-specific survival for responders and non-responders on (**A**) CE-CT and (**B**) 2-[^18^F]FDG-PET/CT on the first follow-up scans.

Kaplan–Meier plots for PFS and DSS for responders and non-responders on CE-CT and 2-[^18^F]FDG-PET/CT are presented in Figs. 1 and 2, respectively. Two-year PFS for responders vs. non-responders by CE-CT was 54.2% vs. 46.0%, compared with 59.1% vs. 14.3% by 2-[^18^F]FDG-PET/CT (Table 3). For patients with bone-only disease, 2-year PFS for responders vs. non-responders by CE-CT was 100% vs. 61.9%, compared with 81.3% vs. 28.6% by 2-[^18^F]FDG-PET/CT.

**Table 3:** Two- and four-year progression-free- and disease-specific survival for responders vs. non-responders and progression vs. non-progression on CE-CT and 2-[^18^F]FDG-PET/CT (N = 87).

	Progression-free survival (95% CI)		Disease-specific survival (95% CI)	
	2-year (%)	4-year (%)	2-year (%)	4-year (%)
All	48.3 (37.5–58.3)	26.5 (15.8–38.3)	79.3 (69.0–86.0)	41.2 (28.4–53.5)
CE-CT				
Responders	54.2 (32.7–71.4)	41.7 (19.9–62.2)	83.3 (61.5–93.4)	50.0 (24.9–71.4)
Non-responders	46.0 (33.5–57.7)	20.0 (8.9–34.2)	77.8 (65.4–86.2)	37.5 (23.1–51.8)
2-[^18^F]FDG-PET/CT				
Responders	59.1 (46.3–69.8)	31.8 (18.3–46.2)	84.9 (73.7–91.5)	46.6 (30.9–60.9)
Non-responders	14.3 (3.6–32.1)	9.50 (1.6–26.1)	61.9 (38.1–78.8)	24.5 (7.73–46.2)
CE-CT				
Non-progression	50.0 (38.9–60.1)	27.4 (16.4–39.5)	81.0 (70.8–87.9)	43.5 (30.3–56.0)
Progression	N/A	N/A	33.3 (0.9–77.4)	N/A
2-[^18^F]FDG-PET/CT				
Non-progression	53.2 (41.6–63.4)	29.1 (17.5–41.8)	82.3 (71.9–89.1)	46.3 (32.3–59.1)
Progression	N/A	N/A	50.0 (15.2–77.5)	N/A

*CI* confidence interval;

Responders = complete (metabolic) response + partial (metabolic) response.

Non-responders = stable (metabolic) disease + progressive (metabolic) disease.

Progression = progressive (metabolic) disease.

Non-progression = complete (metabolic) response + partial (metabolic) response + stable (metabolic) disease.

Only three patients (3/87, 3.45%) were classified with PD on CE-CT, and eight patients (8/87, 9.20%) with PMD on 2-[^18^F]FDG-PET/CT. Due to these small numbers, we did not have sufficient statistical power to calculate 2-year PFS and DSS for progression vs. non-progression.

In an univariate COX proportional hazard regression, tumor response (responders vs. non-responders) on 2-[^18^F]FDG-PET/CT was significantly associated with both PFS (HR 3.55, P < 0.001) and DSS (HR 2.35, P = 0.008), while no association was found for tumor response on CE-CT (HR 1.63, P = 0.12 for PFS and HR 1.59, P = 0.20 for DSS).

### Agreement on response categorization

Examining all follow-up scans (N = 517), only a fair agreement was observed between response categorization on CE-CT and 2-[^18^F]FDG-PET/CT, with a weighted Kappa of 0.28, as shown in Table 2. The disagreement became even more pronounced in patients with bone-only disease, with a weighted Kappa of 0.13 (n = 138, Supplemental Table 1). The agreement remained fair when dichotomizing tumor response into responders vs. non-responders (Kappa 0.29) or progression vs. non-progression (Kappa 0.35) for both modalities (Supplemental Tables 2, 3). The main differences in response categorization were observed when CE-CT suggested non-response and 2-[^18^F]FDG-PET/CT suggested response (122/237, 51.5%). In 20.4% (57/280) of scans, CE-CT suggested response, and 2-[^18^F]FDG-PET/CT indicated non-response.

## Discussion

This prospective study compared response prediction and agreement between response categorization for CE-CT and 2-[^18^F]FDG-PET/CT when used for response monitoring of patients with MBC. Tumor response on 2-[^18^F]FDG-PET/CT was significantly associated with PFS (HR 3.49, P < 0.001) and DSS (HR 2.35, P = 0.008), while no association was found for tumor response on CE-CT. Further, we found a low concordance between response categorization on the two modalities, with 2-[^18^F]FDG-PET/CT suggesting treatment response in every second scan in which CE-CT suggested non-response.

Strengths of the present study are the prospective study design and the external validity, where treatment effect was monitored in patients from daily clinical practice. Patients served as their own control with a unique opportunity to compare scan results within the same patient. Standardized response evaluation criteria were applied for both modalities. For 2-[^18^F]FDG-PET/CT, PERCIST was considered an established semiquantitative assessment with a significantly higher interrater agreement and reliability than qualitative assessment^11,12,22,23^.

However, the single-center observational design limits the generalizability and evaluation of patient-related benefits such as overall survival and quality of life. Further, PERCIST was used for response categorization on 2-[^18^F]FDG-PET/CT by introducing the nadir level of SULpeak before reaching an international consensus. The follow-up of patients in this study changed during follow-up time. At first, treatment response was assessed and acted upon by CE-CT only, but after the end of follow-up in the clinical trial (NCT03358589), most patients switched to response monitoring by 2-[^18^F]FDG-PET/CT. This may have influenced the study results due to evident differences in the timing of detection of progression, leading to changes in medical treatment^9,13,14^. However, the cross-over in the follow-up method was similar for all patients and may not have favored any of the response groups.

We found a low concordance between response categorization between the two modalities. Other studies have compared response categorization by CE-CT and 2-[^18^F]FDG-PET/CT and found 2-[^18^F]FDG-PET/CT to better differentiate response (PMR and CMR) from non-response (SMD and PMD)^9,13^. These findings indicate that 2-[^18^F]FDG-PET/CT provides an early and reliable indication of treatment efficacy compared with CE-CT (Table 1) since it correlates better with PFS and DSS.

One explanation for the differences in response categorization between the two diagnostic modalities could be the high prevalence of bone metastases (approx. 75%) comparable with previous findings^2,24^. Bone lesions are difficult to detect by CE-CT and may challenge its capability to distinguish between tumor response and tumor progression in such lesions^10,12,13^. As observed in this study, the disagreement in response categorization between the two modalities increased when analyzing patients with bone-only disease (Supplemental Table 1).

In daily clinical practice, imaging is a tool for supporting decision-making where disease progression leads to a change of treatment. Stable disease is often considered a favorable outcome (disease control), leading to the continuation of ongoing treatment. In this and other studies^9,25^, stable disease occurred more often by CE-CT than 2-[^18^F]FDG-PET/CT. However, we found no difference in survival when analyzing the disease control rate by moving patients with stable disease on CE-CT from the non-response group to the non-progressive group, indicating stable disease to be less favorable. Therefore, more sensitive imaging such as 2-[^18^F]FDG-PET/CT may have the potential to improve clinical decision-making, optimize the timing of treatment alterations, and avoid treating patients with ineffective toxic and expensive treatments. The early separation of responders and non-responders for 2-[^18^F]FDG-PET/CT may also influence future suggestions for length of monitoring intervals due to higher clinical confidence in responses assessed by 2-[^18^F]FDG-PET/CT than CE-CT.

Treatment response is an established surrogate for treatment efficacy in clinical trials. This application is another advantage for 2-[^18^F]FDG-PET/CT with evidence of its superiority in differentiating responders from non-responders. Additionally, 2-[^18^F]FDG-PET/CT and PERCIST classify more patients with measurable disease than CE-CT and RECIST^9,13^. An objective measure of disease is a common prerequisite for enrollment in clinical trials evaluating new treatments, and the higher sensitivity of 2-[^18^F]FDG-PET/CT may enable a higher level of evidence for new treatments.

### Perspectives

Current evidence suggests several advantages of applying 2-[^18^F]FDG-PET/CT for response monitoring in MBC, but will response monitoring by 2-[^18^F]FDG-PET/CT positively impact overall survival and quality of life for patients with MBC compared with conventional CT? The question can only be answered by a multi-center randomized controlled trial and is the perspective for future research.

## Conclusion

In this prospective observational study, we found 2-[^18^F]FDG-PET/CT to be a better predictor of progression-free and disease-specific survival than CE-CT when used to monitor treatment effects in women with metastatic breast cancer. In addition, we found low concordance between response categorization between CE-CT and 2-[^18^F]FDG-PET/CT. Further studies comparing the two modalities for patient-related benefits such as overall survival and quality of life are warranted.

## Supplementary Information

Supplementary Information.

## Data Availability

The datasets generated and analyzed during the current study are available from the corresponding author upon reasonable request.

## References

[1] Cardoso F, Paluch-Shimon S, Senkus E, Curigliano G, Aapro MS, André F (2020). 5th ESO-ESMO international consensus guidelines for advanced breast cancer (ABC 5)(†). Ann. Oncol..

[2] Harbeck N, Penault-Llorca F, Cortes J, Gnant M, Houssami N, Poortmans P (2019). Breast cancer. Nat. Rev. Dis. Primers.

[3] Waks AG, Winer EP (2019). Breast cancer treatment: A review. WaksJama.

[4] NCCN Clinical Practice Guidelines: Breast Cancer. 2021. https://www.nccn.org/professionals/physician_gls/pdf/breast.pdf. Accessed 08 June 2021.

[5] Eisenhauer EA, Therasse P, Bogaerts J, Schwartz LH, Sargent D, Ford R (2009). New response evaluation criteria in solid tumours: Revised RECIST guideline (version 11). Eur. J. Cancer (Oxford, England; 1990).

[6] Burzykowski T, Buyse M, Piccart-Gebhart MJ, Sledge G, Carmichael J, Lück HJ (2008). Evaluation of tumor response, disease control, progression-free survival, and time to progression as potential surrogate end points in metastatic breast cancer. J. Clin. Oncol..

[7] Wahl RL, Jacene H, Kasamon Y, Lodge MA (2009). From RECIST to PERCIST: Evolving considerations for PET response criteria in solid tumors. J. Nucl. Med..

[8] Groheux D (2018). Role of fludeoxyglucose in breast cancer: Treatment response. PET Clin..

[9] Riedl CC, Pinker K, Ulaner GA, Ong LT, Baltzer P, Jochelson MS (2017). Comparison of FDG-PET/CT and contrast-enhanced CT for monitoring therapy response in patients with metastatic breast cancer. Eur. J. Nucl. Med. Mol. Imaging.

[10] Hildebrandt MG, Gerke O, Baun C, Falch K, Hansen JA, Farahani ZA (2016). [18F]Fluorodeoxyglucose (FDG)-positron emission tomography (PET)/computed tomography (CT) in suspected recurrent breast cancer: A prospective comparative study of dual-time-point FDG-PET/CT, contrast-enhanced CT, and bone scintigraphy. J. Clin. Oncol..

[11] Vogsen M, Bulow JL, Ljungstrom L, Oltmann HR, Alamdari TA, Naghavi-Behzad M (2021). FDG-PET/CT for response monitoring in metastatic breast cancer: The feasibility and benefits of applying PERCIST. Diagnostics (Basel).

[12] Hildebrandt MG, Naghavi-Behzad M, Vogsen M (2022). A role of FDG-PET/CT for response evaluation in metastatic breast cancer?. Semin. Nucl. Med..

[13] Vogsen M, Harbo F, Jakobsen NM, Nissen HJ, Dahlsgaard-Wallenius SE, Gerke O (2022). Response monitoring in metastatic breast cancer—a prospective study comparing (18)F-FDG PET/CT with conventional CT. J. Nucl. Med..

[14] Naghavi-Behzad M, Vogsen M, Vester RM, Olsen MMB, Oltmann H, Braad PE (2022). Response monitoring in metastatic breast cancer: A comparison of survival times between FDG-PET/CT and CE-CT. Br. J. Cancer.

[15] von Elm E, Altman DG, Egger M, Pocock SJ, Gøtzsche PC, Vandenbroucke JP (2007). The strengthening the reporting of observational studies in epidemiology (STROBE) statement: Guidelines for reporting observational studies. Lancet (London, England).

[16] Vogsen M, Jensen JD, Christensen IY, Gerke O, Jylling AMB, Larsen LB (2021). FDG-PET/CT in high-risk primary breast cancer-a prospective study of stage migration and clinical impact. Breast Cancer Res. Treat..

[17] Vogsen M, Jensen JD, Gerke O, Jylling AMB, Asmussen JT, Christensen IY (2021). Benefits and harms of implementing [(18)F]FDG-PET/CT for diagnosing recurrent breast cancer: A prospective clinical study. EJNMMI Res..

[18] Boellaard R, Delgado-Bolton R, Oyen WJ, Giammarile F, Tatsch K, Eschner W (2015). FDG PET/CT: EANM procedure guidelines for tumour imaging: Version 20. Eur. J. Nucl. Med. Mol. Imaging.

[19] Jh O, Lodge MA, Wahl RL (2016). Practical PERCIST: A simplified guide to PET response criteria in solid tumors 1.0. Radiology.

[20] Jager KJ, van Dijk PC, Zoccali C, Dekker FW (2008). The analysis of survival data: The Kaplan–Meier method. Kidney Int..

[21] Landis JR, Koch GG (1977). The measurement of observer agreement for categorical data. Biometrics.

[22] Sørensen JS, Vilstrup MH, Holm J, Vogsen M, Bülow JL, Ljungstrøm L (2020). Interrater agreement and reliability of PERCIST and visual assessment when using 18F-FDG-PET/CT for response monitoring of metastatic breast cancer. Diagnostics (Basel).

[23] Fledelius J, Khalil A, Hjorthaug K, Frokiaer J (2016). Inter-observer agreement improves with PERCIST 1.0 as opposed to qualitative evaluation in non-small cell lung cancer patients evaluated with F-18-FDG PET/CT early in the course of chemo-radiotherapy. EJNMMI Res..

[24] Buonomo OC, Caredda E, Portarena I, Vanni G, Orlandi A, Bagni C (2017). New insights into the metastatic behavior after breast cancer surgery, according to well-established clinicopathological variables and molecular subtypes. PLoS One.

[25] Naghavi-Behzad M, Oltmann HR, Alamdari TA, Bülow JL, Ljungstrøm L, Braad PE (2021). Clinical impact of FDG-PET/CT compared with CE-CT in response monitoring of metastatic breast cancer. Cancers (Basel).

