# Supplementary material for: 2-[18F]FDG-PET/CT is a better predictor of survival than conventional CT: a prospective study of response monitoring in metastatic breast cancer
Source: Sci Rep. 2023 Apr 5;13:5552. doi: 10.1038/s41598-023-32727-w (PMC10076261; doi:10.1038/s41598-023-32727-w)
Supplement: Supplementary file 1 — Supplementary Information. [file 41598_2023_32727_MOESM1_ESM.docx]

**Supplemental Tables**

| **Supplemental Table 1** Distribution and agreement between response categorization according to CE-CT and 2-[^18^F]FDG-PET/CT for patients with bone-only disease (N = 138) | | | | | | |
| --- | --- | --- | --- | --- | --- | --- |
|  | **CE-CT** | | | | | |
|  |  | CR | PR | SD | PD | **Total** |
| **2-[^18^F]FDG-PET/CT** | CMR | **0** | 18 | 14 | 1 | 33 |
|  | PMR | 0 | **19** | 31 | 2 | 52 |
|  | SMD | 0 | 0 | **14** | 0 | 14 |
|  | PMD | 0 | 14 | 18 | **7** | 39 |
|  | **Total** | 0 | 51 | 77 | 10 | **138** |
|  | Agreement 29.0% | | Weighted Kappa 0.13 | | Std. Error 0.04 | |
| Abbreviations: C(M)R, complete (metabolic) response; P(M)R, partial (metabolic) response; S(M)D, stable (metabolic) disease; P(M)D, progressive (metabolic) disease | | | | | | |

| **Supplemental Table 2** Distribution and agreement between responders and non-responders on CE-CT and 2-[^18^F]FDG-PET/CT in 87 patients with 517 follow-up scans. | | | | |
| --- | --- | --- | --- | --- |
|  | **CE-CT** | | | |
| **2-[^18^F]FDG-PET/CT** |  | Responders | Non-responders | **Total** |
|  | Responders | **223** | 122 | 345 |
|  | Non-responders | 57 | **115** | 172 |
|  | **Total** | 280 | 237 | **517** |
|  | Agreement 65.4% | | Kappa 0.29 | Std. Error 0.04 |
| Abbreviations:  Responders = complete (metabolic) response + partial (metabolic) response  Non-responders = stable (metabolic) disease + progressive (metabolic) disease | | | | |

| **Supplemental Table 3** Distribution and agreement between progression and non-progression on CE-CT and 2-[^18^F]FDG-PET/CT in 87 patients with 517 follow-up scans. | | | | |
| --- | --- | --- | --- | --- |
|  | **CE-CT** | | | |
| **2-[^18^F]FDG-PET/CT** |  | Progression | Non-progression | **Total** |
|  | Progression | **40** | 96 | 136 |
|  | Non-progression | 7 | **374** | 381 |
|  | **Total** | 47 | 470 | **517** |
|  | Agreement 80.1% | | Kappa 0.35 | Std. Error 0.04 |
| Progression = progressive (metabolic) disease  Non-progression = complete (metabolic) response + partial (metabolic) response + stable (metabolic) disease | | | | |
